# Cardiotoxicity prevention in thoracic radiotherapy: The effect of different melatonin doses on the level of oxidation markers -in vivo animal study

**DOI:** 10.1016/j.toxrep.2025.102030

**Published:** 2025-04-17

**Authors:** Ecem Demir, Karolin Yanar, Pınar Atukeren, Serbay Ozkan, Gözde Erkanlı Şentürk, Melike Ülker, Şefika Arzu Ergen, Songül Karaçam, Fazilet Öner Dinçbaş

**Affiliations:** a Başakşehir Çam and Sakura City Hospital, Department of Radiation Oncology, Istanbul, Turkey; b Istanbul University-Cerrahpaşa, Cerrahpaşa Faculty of Medicine, Department of Biochemistry, Istanbul, Turkey; c Izmir Katipcelebi University Faculty of Medicine, Department of Histology and Embryology, Istanbul, Turkey; d Istanbul University-Cerrahpaşa, Cerrahpaşa Faculty of Medicine, Department of Histology and Embryology, Istanbul, Turkey; e Tekirdag Dr. Ismail Fehmi Cumalioglu City Hospital, Department of Thoracic Surgery, Tekirdag, Turkey; f Istanbul University-Cerrahpaşa, Cerrahpaşa Faculty of Medicine, Department of Radiation Oncology, Istanbul, Turkey; g Istanbul University-Cerrahpaşa, Vocational School of Health Services, Radiotherapy Program, Istanbul, Turkey

**Keywords:** Melatonin, RT-induced hearth disease, Cardiotoxicity, Rat, Antioxidant

## Abstract

**Introduction:**

Radiation-induced cardiotoxicity (RIC) is a significant adverse effect of thoracic radiotherapy (RT), leading to oxidative stress, inflammation, endothelial dysfunction, myocardial fibrosis, vascular damage, and cardiac dysfunction. Melatonin (MLT), a potent antioxidant and radioprotective agent, has been suggested to mitigate these effects. This study aims to evaluate the optimal dosage of MLT for cardioprotection following RT in a rat model.

**Materials and methods:**

Forty-five adult male Sprague-Dawley rats were divided into five groups. The control group received 1 mL saline solution and sham irradiation. The RT-only group received 12 Gy RT in a single fraction with saline. Three experimental groups received the same RT dose with MLT at 100 mg/kg, 50 mg/kg, or 5 mg/kg. Eight weeks post-irradiation, protein oxidation, lipid peroxidation, glycoxidation, non-enzymatic redox homeostasis biomarkers, and histological changes in heart tissues were examined.

**Results:**

In MLT-treated groups, 5 mg/kg dose was found to be more effective in preventing protein oxidation and lipid peroxidation. The levels of advanced glycation end products were significantly lower in 5 mg/kg and 50 mg/kg MLT groups compared to the RT only group, whereas no difference was found at the high dose (100 mg/kg). When Cu-Zn superoxide dismutase activity, iron ion reducing antioxidant power and total thiol groups were evaluated, we found that 5 mg/kg MLT caused a significant increase in these antioxidant parameters, while 50 mg/kg and 100 mg/kg dose caused significant increase in superoxide dismutase. Evaluation heart tissues showed that the RIC was significantly lower in all MLT-treated groups.

**Conclusion:**

5 mg/kg melatonin reduces oxidative markers and RIC in rats, indicating its potential as a low-dose cardioprotective agent after RT.

## Highlights

• **Optimal Dose for Oxidative Stress:** A 5 mg/kg dose of melatonin effectively reduced oxidative markers like protein and lipid peroxidation and advanced glycation end products. It outperformed higher doses, demonstrating its potential as a low dose radioprotective agent.
• **Antioxidant Activity Boost:** Melatonin at 5 mg/kg significantly enhanced antioxidant defenses, increasing Cu-Zn superoxide dismutase activity and total thiol groups. This highlights its role in maintaining redox balance during radiotherapy.
• **Reduced Cardiac Damage:** All melatonin-treated groups showed reduced cardiac damage, with 5 mg/kg providing the strongest protection. This suggests melatonin's effectiveness in preventing radiotherapy-induced cardiotoxicity.

## Graphical Abstract

**Figure:**
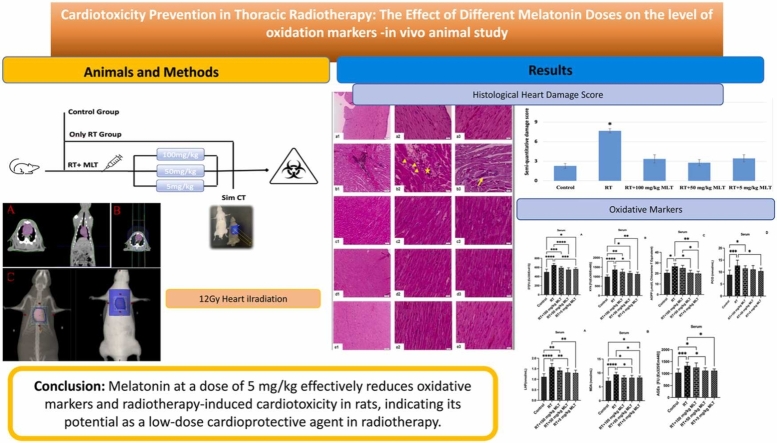

## 1 Introduction

According to GLOBOCAN 2022 data, lung cancer is leading causes of death worldwide with 2.5 million cases in the year 2022 [1]. Based on the available literature, approximately 50–76 % of patients diagnosed with lung cancer receive thoracic radiotherapy (RT) as part of their treatment regimen. This percentage varies based on factors such as disease stage and overall health [2]. Specific studies have shown that in the context of non-small cell lung cancer (NSCLC), which is the most common histology of lung cancer, a significant percentage is indicated for thoracic RT, particularly in advanced stages where surgical options may not be viable [3].

While thoracic RT remains a cornerstone of lung cancer therapy, the close anatomical proximity of the heart to the lungs often places it at risk, leading to an increase in radiation-induced cardiac toxicity (RIC) as radiation directed at thoracic tumors can unintentionally expose cardiac structures and contribute to long-term cardiovascular complications. The prevalence of RIC among lung cancer patients undergoing thoracic radiotherapy (RT) is a significant concern and varies across studies. Research indicates that approximately 20–30 % of patients treated with thoracic RT experience some degree of cardiac complications. In a study with lung cancer patients who were treated with thoracic RT, it was observed that 29 % of patients developed cardiac complications as assessed by myocardial perfusion imaging [4]. In another study, it was noted that after 5 years, patients' absolute risk of cardiac morbidity is 2 % greater, and after 20 years, it is 23 % higher compared to non-irradiated patients [5]. RIC manifests in several detrimental forms, including heart failure, myocardial ischemia, arrhythmias, and infarctions, which can adversely affect patient outcomes and survival [6]. The underlying mechanism for this damage is primarily attributed to oxidative stress induced by ionizing radiation, which generates reactive oxygen species (ROS) and leads to cellular injury [7]. Consequently, there has been a growing interest in identifying protective agents that could potentially mitigate these effects.

Melatonin, a pleiotropic hormone known for its strong antioxidant properties, has been highlighted in various studies for its potential role as a radioprotective agent. Specifically, it has been shown to counteract oxidative stress and promote cellular survival in various biological contexts [7], [8]. Research indicates that melatonin exerts its protective effects through its potent scavenging of free radicals and modulation of autophagy, which is critical for maintaining cellular homeostasis [9]. The protective efficacy of melatonin appears to be dose-dependent; however, the optimal dosage for cardioprotection in the context of thoracic RT is still poorly defined [10]. Previous investigations have shown mixed results regarding the appropriate administration dosing strategies for melatonin to maximize its cardioprotective effects, highlighting the need for further research to elucidate these variables.

In a comparative context, the efficacy of melatonin needs to be evaluated alongside other antioxidants, such as vitamin E and N-acetylcysteine (NAC), known for their protective roles against oxidative stress. Research indicates that these antioxidants also offer substantial protection against radiation-induced cellular damage, yet they vary in mechanism and effectiveness [11], [12]. For example, vitamin E acts primarily by interrupting lipid peroxidation, while NAC replenishes intracellular glutathione levels and enhances detoxification pathways [11]. Such comparative studies could elucidate the most effective antioxidant for preventing cardiac damage resultant from RT, thus optimizing treatment for lung cancer patients with a high cardiac risk.

This study aims to bridge the existing knowledge gap by systematically evaluating the dose-dependent efficacy of melatonin in safeguarding against RT-induced cardiac injury using an in vivo rat model. Through meticulous assessment of oxidative stress markers and histological alterations in heart tissue, we seek to determine the ideal melatonin dosage that confers maximum cardioprotection. Our findings will not only improve understanding of melatonin’s role within the context of thoracic RT but will also provide important insights into optimizing treatment strategies to enhance patient outcomes.

## 2 Material-method

### 2.1 Rats

Experiments were performed in Bezmialem Foundation University Experimental Application and Research Center (14.09.2022-E.77287). A total of 45 adult male Sprague Dawley rats (200–300 g) were used. During the experiment, rats were housed in standard plastic rat cages (23 ± 2°C room temperature, 55 ± 10 % relative humidity, 12 h day/night light period) and fed ad-libitum standard rat chow and fresh drinking water.

### 2.2 Radiotherapy technique

**Fig. 1:**
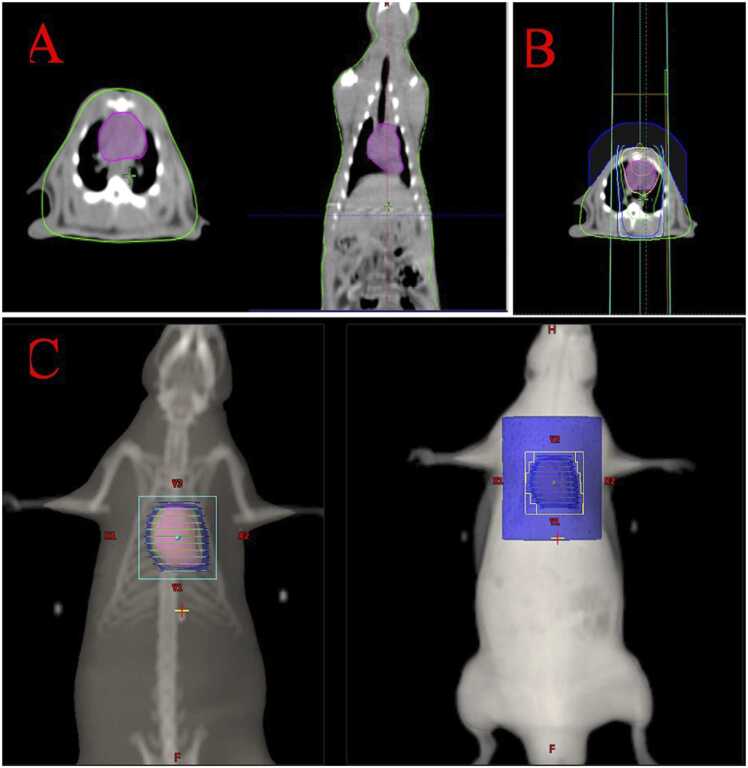
A. Contoured volume of the heart in axial and coronal planes B. and C. 3D conformal radiotherapy demonstration of the treatment volume in axial and coronal sections.

Animals received intraperitoneal injections of ketamine hydrochloride (Ketalar1; EWL Eczacıbaşı Warner Lambert İlaç ¸ Sanayi ve Ticaret A.Ş., İstanbul) at doses of 60–90 mg/kg and xylazine hydrochloride (Rompun 2 % Bayer Kimya San. Ltd. Şti., İstanbul, Türkiye) at doses of 6–10 mg/kg. The animals were then placed in the supine position on a tissue-equivalent foam tray with their extremities taped and simulated computed tomography (CT) was performed. The whole heart volume was contoured on CT images and a 3D conformal RT (3D-CRT) treatment plan was made. The rats were then irradiated with an X-ray linear accelerator device (Rapid Arc, Varian Medical Systems, Palo Alto, USA) using a 6 megavolt (MV) photon beam at 100 cm from the source to the surface. The whole heart volume received 12 Gy X-rays in a single fraction at a dose rate of 300 monitor units (MU). A 1 cm thick elastic-gel bolus was placed 1 cm above the thorax to maximise the cardiac dose [13], [14], [15]. Contoured volume of the heart in axial and coronal planes is demonstrated in Fig. 1.

Rats were randomly divided into 5 groups: The first group was the control group and only 1 mL saline solution was injected intraperitoneally, and they were kept in the irradiation position for the same fraction duration and under the same conditions (sham irradiation). The second group was injected with 1 mL saline solution and 30 minutes later the rats were exposed to radiation which will be detailed in the following section. For treatment groups, melatonin (N-acetyl-5-methyoxytryptamine, Sigma-Aldrich, USA) was dissolved in ethanol and diluted in 0.9 % saline solution to a concentration of 10 mg/mL. In the third group, melatonin dose was 100 mg/kg [16], in the fourth group the dose of melatonin was 50 mg/kg, and in the fifth group 5 mg/kg [8] melatonin were applied 30 min before RT intraperitoneally. Numerous studies in the literature have evaluated the effect of specific melatonin doses in mitigating radiation-induced cardiotoxicity (RIC). However, very few have compared the effects of different doses on RIC. Therefore, our study aims to help researchers identify the minimum effective dose for optimal mitigation. We designed our experiment to compare the lowest dose used 5 mg/kg, the highest dose 100 mg/kg, and an intermediate dose of 50 mg/kg [17].

### 2.3 Collection of rat plasma and serum samples

The duration of the experiment was determined as 8 weeks based on previous RIC studies [6], [18]. At 8th week, the animals were euthanized by exsanguination following anesthesia. Blood sampling was performed without fasting, with samples collected via cardiac puncture. Blood was collected into heparinised collection tubes containing EDTA and serum tubes. Serum and plasma samples obtained by centrifugation at 2000 rpm for 10 min using a chilled centrifuge were pipetted into Eppendorf tubes and stored at −20°C until evaluation in 1 month. Protein oxidation, lipid peroxidation, glycoxidation, enzymatic and non-enzymatic redox homeostasis biomarker levels were analysed in serum and plasma samples. Lipid hydroperoxide and malondialdehyde were analysed in plasma samples whereas all other markers were analysed in serum samples. The evaluated parameters are given in Table 1.

**Table 1:** The effect of intraperitoneal administration of different melatonin doses on certain biochemical parameters in male Sprague Dawley rats.

**Parameter** (Mean±SD)	**Group 1 (Control)**	**Group 2 (RT)**	**Group 3 (MLT dose, 100 mg/kg)**	**Group 4 (MLT dose, 50 mg/kg)**	**Group 5 (MLT dose, 5 mg/kg)**
**Protein oxidation biomarkers**					
Dityrozine (nmol/mg)	497,8 ± 63,22	650,8 ± 35,21	592,4 ± 26,91	550,1 ± 40,71	564,9 ± 29,57
Kinurenin (µmol/L)	994,3 ± 98,37	1370 ± 200,6	1250 ± 143,0	1188 ± 95,57	1135 ± 101,2
Advanced Oxidation Protein Products (µmol/L)	20,53 ± 2,57	26,72 ± 2,60	25,10 ± 2,57	20,58 ± 2,45	19,80 ± 2,24
Protein Carbonyl (nmol/mg)	8,85 ± 1,99	12,71 ± 2,75	11,52 ± 1,10	11,11 ± 1,60	10,39 ± 1,24
**Lipid peroxidation biomarkers**					
Lipid hydroperoxide (µmol/L)	0,5 ± 0,17	0,80 ± 0,18	0,59 ± 0,22	0,51 ± 0,12	0,44 ± 0,19
Malondialdehyde (µmol/L)	5603 ± 0,6276	10,19 ± 1729	7913 ± 1118	7015 ± 0,6617	7048 ± 0,8838
**Glycoxidation biomarker**					
Advanced Glycation End Products (µg/mL)	1037 ± 154,9	1325 ± 147,6	1254 ± 191,9	1122 ± 120,7	1121 ± 73,93
**Non-enzymatic biomarker of redox homeostasis**					
Total Thiol Groups (µmol/L)	29,60 ± 6533	21,51 ± 2498	25,99 ± 2859	26,60 ± 4092	29,86 ± 8087
Iron Ion Reducing Antioxidant Power (µmol Fe²⁺/L)	25,15 ± 2936	21,07 ± 0,9704	21,65 ± 1514	22,50 ± 1540	24,11 ± 1961
**Enzymatic biomarker of redox homeostasis**					
Cu, Zn-Superoxide Dismutase (U/mL)	6128 ± 0,6736	3697 ± 0,7939	4975 ± 0,8602	4907 ± 0,4799	5390 ± 0,9342

Male Sprague Dawley rats (n = 45) were given intraperitoneally different doses of melatonin before 30 min of 12Gy-1fraction thoracic RT. After 8 weeks, oxidation and redox biochemical parameters are evaluated.

### 2.4 Histological evaluation

Histologically, myocardial tissues of the rats were examined with Haematoxylin Eosin staining. Fibrosis rates in heart tissue and the protective effect of melatonin for cardiac fibrosis were evaluated by histological scoring. The heart tissue obtained from mediastinal dissection was fixed in 10 % buffered formaldehyde for 24 hours. After routine tissue processing was completed, 5 μm transverse sections were taken to show the ventricular walls and interventricular septum of the heart. The sections obtained were stained with haematoxylin and eosin. Then, at 40x magnification, 10 randomly determined areas from the endocardial, myocardial and epicardial layers of the heart were evaluated with the semi-quantitative scoring system [19].

Based on established scoring systems described by Gürses et al., and Krishnamurthy et al., 4 key parameters—inflammatory cell infiltration, perivascular edema, cardiomyocyte injury, and fibrosis—were each evaluated using a semi-quantitative scale from 0 (none) to 3 (intense)[19], [20]. A score of 0 indicates an absence of the respective pathological finding, whereas a score of 3 denotes the highest severity. Intermediate values (1 or 2) reflect mild to moderate changes, respectively. This standardized approach facilitates a consistent and comparative assessment of the extent of cardiac tissue damage across experimental groups.

### 2.5 Statistical analyses

The suitability of the variables for normal distribution was analysed by Shapiro-Wilk test. Descriptive analyses were given using median, mean and standard deviations for normally distributed variables. Student's t test was used for variables suitable for normal distribution, and Mann-Whitney *U* test was used for variables that did not show normal distribution.

Biochemical parameters of the treatment and control groups were compared using one-way analysis of variance (ANOVA) and Tukey's multiple comparison test. All data are presented as mean ± standard deviation (SD) and median. Since pathological scores were ordinal, differences in pathological findings between the study groups were analysed using the Kruskal Wallis test. When a statistically significant difference was observed in general, pairwise comparisons were made with Mann-Whitney *U* test. Differences between the groups were accepted as significant for p-value < 0.05. Statistical analyses were performed with GraphPad Prism 5.0 (GraphPad Software, Inc., San Diego, CA). Bonferroni correction was used for multiple comparisons.

## 3 Results

### 3.1 Protein oxidation biomarkers

The levels of all protein oxidation biomarkers (advanced oxidation protein products, dityrozine, kinurenin and protein carbonyl) were found to be significantly higher in the RT group (group 2) compared to the control group (p < 0.05; p < 0.0001; p < 0.0001; p < 0.001, respectively). All biomarkers were higher in RT groups than RT+ 5 mg/kg melatonin group (p < 0.001; p < 0.01; p < 0.01; p < 0.05, respectively). When RT+ 5 mg/kg melatonin group (group 5) was compared with the control group (group 1), a significant difference was found in dityrosine biomarker levels (p < 0.05), whereas there was no difference between the groups in kynurenine and advanced oxidation protein products. Dityrosine levels remained significantly higher in this treatment group. In general, it was observed that 5 mg/kg melatonin administration was largely effective in preventing protein oxidation (Supplementary Fig. 1).

In the group administered 100 mg/kg melatonin (group 3), dityrosine, kynurenine and protein carbonyl oxidase values were significantly higher than the control group (group 1) (p < 0.001; p < 0.01; p < 0.05, respectively). In addition, advanced oxidation protein product levels of 100 mg/kg melatonin group (group 3) were found to be higher than 5 mg/kg melatonin group (group 5) (p < 0.05) (Table 1).

### 3.2 Lipid peroxidation biomarkers

The levels of both lipid peroxidation biomarkers (plasma lipid hydroperoxide, malondialdehyde) were significantly higher in the RT group (group 2) compared to the control group (p < 0.01; p < 0.0001, respectively), similarly significantly higher compared to the RT+ 5 mg/kg melatonin group (p < 0.001; p < 0.05), significantly higher when compared with RT+ 50 mg/kg melatonin group (p < 0.01; p < 0.05, respectively), and no significant difference was found when compared with RT+ 100 mg/kg melatonin group (p > 0.05). In addition, malondialdehyde levels were significantly higher in the RT+ 100 mg/kg melatonin group compared to the control group (p < 0.01)(Table 1) (Supplementary Fig. 2).

### 3.3 Advanced glycation products

Advanced glycation products were significantly higher (p < 0.001) in the RT group (group 2) compared to the control group. Advanced glycation product levels were significantly lower in the low dose and medium dose melatonin group compared to the RT group, whereas no significant difference was found at high dose (Table 1) (Supplementary Fig. 3).

### 3.4 Antioxidant parameters

All three antioxidant parameters were significantly lower in the RT group (group 2) compared to the non-RT group (group 1) (p < 0.05). It was observed that low dose melatonin caused a significant increase in antioxidant parameters, medium level melatonin and high-level melatonin application caused an increase in superoxide dismutase activity. Cu, Zn superoxide dismutase activity of the control group was higher compared to all three doses, and iron ion reducing antioxidant levels were higher than the highest dose (Table 1) (Supplementary Fig. 4).

### 3.5 Histopathologic evaluation of heart tissues

**Fig. 2:**
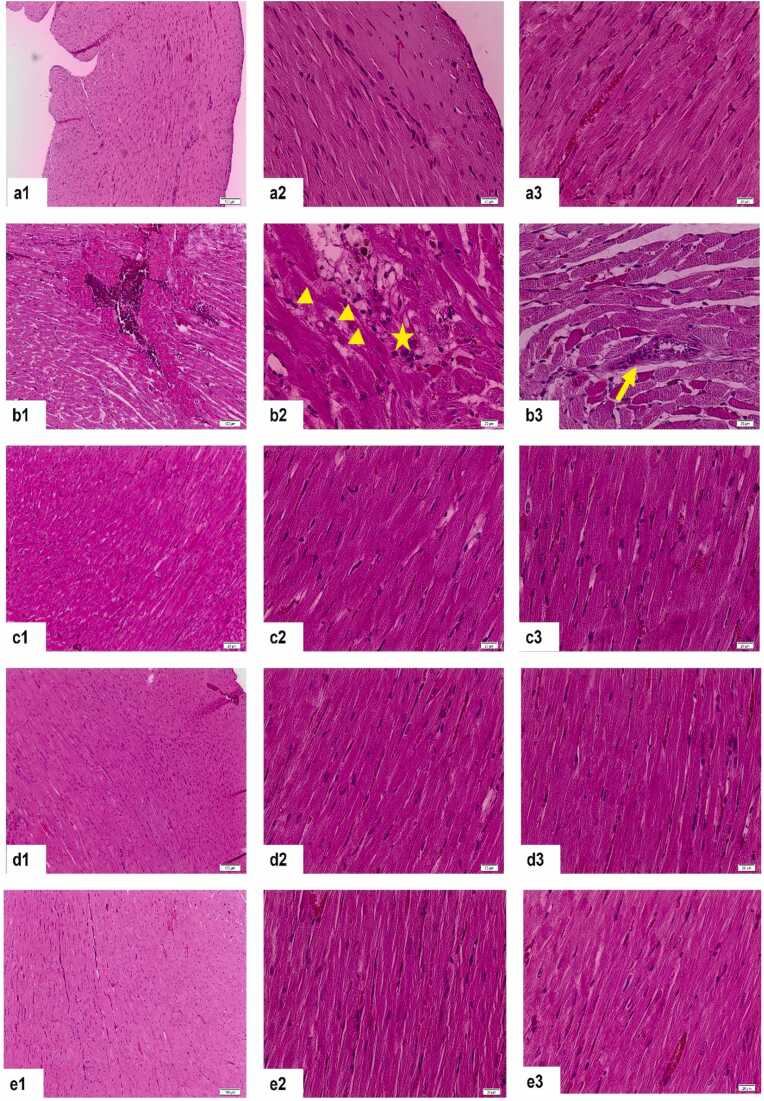
Representative images of H+E-stained heart tissues (group 1 (a), group 2 (b), group 3 (c), group 4 (d) and group 5 (e). Asterisk: inflammatory cell infiltration; arrowhead: cardiomyocyte injury, arrow: perivascular edema.

**Fig. 3:**
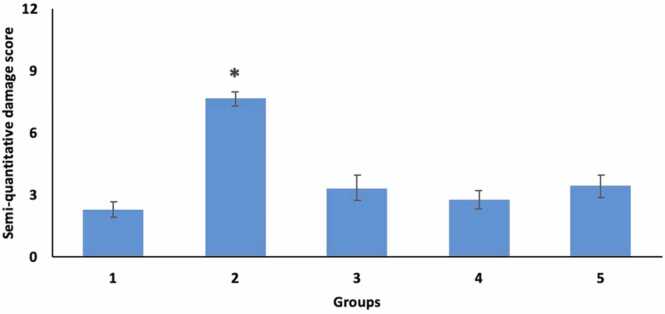
Graphical representation of semiquantitative heart damage scoring results. *p < 0.05 versus all other groups.

We performed a semi-quantitative evaluation of haematoxylin and eosin-stained heart tissues by examining four key parameters—inflammatory cell infiltration, perivascular edema, cardiomyocyte injury, and fibrosis—each scored on a scale from 0 (none) to 3 (intense). The mean scores for each treatment group are presented in Table 2. Our findings showed that the RT group (Group 2) exhibited a significantly higher level of damage compared to the control group (p < 0.0001). On the other hand, it was found that this damage was significantly improved in the treatment groups (Groups 3, 4 and 5) and there was no statistical difference between them and the control group (Fig. 2, Fig. 3).

**Table 2:** Mean inflammatory cell infiltration, perivascular edema, cardiomocyte damage and fibrosis values of heart tissues stained with H+E between groups.

**Parameter** (Mean±SD)	**Group 1 (Control)**	**Group 2 (RT)**	**Group 3 (MLT dose, 100 mg/kg)**	**Group 4 (MLT dose, 50 mg/kg)**	**Group 5 (MLT dose, 5 mg/kg)**
Inflammatory cell infiltration	0,66 ± 0,86	1,77 ± 0,61	0,35 ± 0,62	0,22 ± 0,44	0,55 ± 1,01
Perivascular edema	0,55 ± 0,72	2,33 ± 0,66	0,57 ± 0,53	0,33 ± 0,70	0,66 ± 0,70
Cardiomyocyte injury	1,05 ± 0,16	2,50 ± 0,50	1,57 ± 0,78	1,22 ± 0,61	1,33 ± 0,66
Fibrosis	0	1,05 ± 0,16	0,85 ± 0,37	1,00 ± 0	0,88 ± 0,33
Total Score	2.28 ± 0,38	7.67 ± 0,33	3.36 ± 0,64	2.78 ± 0,47	3.44 ± 0,55

## 4 Discussion

### 4.1 Comparison with previous findings

Our findings contribute to the growing body of research exploring the optimal dosage of melatonin (MLT) for radioprotection and antioxidant activity. While multiple studies have investigated the effects of MLT at various doses in different tissues, few have directly compared these doses to pinpoint the ideal therapeutic range. For instance, Tahamtan et al. [16] demonstrated that 100 mg/kg MLT was effective in mitigating radiation-induced damage in rat lung tissue, whereas Koc et al. [8] showed no significant difference in lipid peroxidation (MDA) and antioxidant enzyme (SOD) levels when comparing 5 and 10 mg/kg MLT in rats receiving 6 Gy whole-body irradiation. In our study, we evaluated 5, 50, and 100 mg/kg MLT and found that the 5 mg/kg dose was just as effective as higher doses in reducing oxidative damage markers, indicating that a lower dose of MLT may suffice to achieve cardioprotection.

### 4.2 Effect of lower and higher doses on cellular level

Evidence suggests that low doses on cellular level primarily exert oncostatic effects and enhance cellular resistance to stress, while higher doses can trigger apoptosis in certain cell types such as cancer cells. At low concentrations, MLT interacts predominantly with specific melatonin receptors located on cell membranes, facilitating signal transduction pathways that promote cytoprotection and inhibit tumor growth. Zhu et al. highlight that low doses of MLT exert oncostatic effects on tumor cell lines through receptor-mediated pathways, distinct from those activated at higher concentrations, thus indicating a nuanced mechanism of action dependent on dosage [9]. This receptor-mediated response can lead to reduced oxidative stress and enhanced cellular survival under adverse conditions, which is significant for the maintenance of cell integrity, especially in the context of proliferative diseases.

In contrast, at higher concentrations, MLT can induce apoptosis in certain cell types through ROS and mitochondrial damage. For example, Girish et al. demonstrate that elevated levels of MLT correlate with increased apoptosis of platelets by ROS-mediated mitochondrial impairment [21]. Additionally, Zhu et al. affirm that while higher doses increase apoptosis, they may also amplify oxidative stress in some cell contexts—underscoring the duality in MLT’s biological effects based on concentration [9]. Moreover, the cellular distribution of MLT at varying doses extrapolates into distinct mechanisms at the subcellular level. Venegas et al. discuss how higher doses lead to saturation in MLT levels within critical organelles like mitochondria and the nucleus, thus limiting its further functional accumulation beyond a certain threshold [22]. The protective effects related to low doses versus the cytotoxic impacts of high doses could thus be tied to this saturation phenomenon, as the rapid cytosolic presence may engage distinct cellular pathways that either promote proliferation or lead to apoptosis. These findings highlight the need for further exploration into personalized cancer treatment protocols that optimize MLT’s therapeutic benefits while minimizing adverse effects, emphasizing the importance of dose precision in clinical applications.

### 4.3 Melatonin's effect on inflammatory markers or signaling pathways

MLT, a bioactive molecule best known for its role in regulating circadian rhythms, has significant influence on various inflammatory markers and signaling pathways across different cellular contexts. The intricate interplay between MLT and inflammation is mediated primarily through the modulation of cytokines, reactive oxygen species (ROS), and specific signaling like JAK/STAT, PI3K/Akt, and MAPK.

**Fig. 4:**
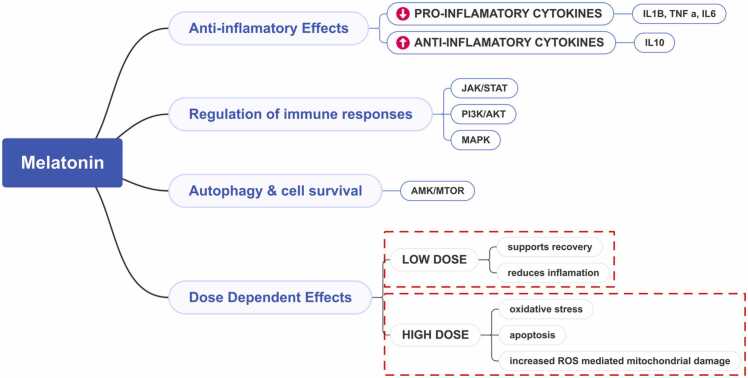
Melatonin’s effect on inflammatory markers and signaling pathways: a graphic demonstration.

One key mechanism by which MLT exerts anti-inflammatory effects is through its ability to reduce pro-inflammatory cytokines like interleukin-1 beta (IL-1β), tumor necrosis factor-alpha (TNF-α), and interleukin-6 (IL-6). It was demonstrated that melatonin can significantly attenuate the inflammatory response in human mesenchymal stem cells by blocking the effects of IL-1β, thereby decreasing the levels of these inflammatory markers and reducing cellular toxicity associated with inflammatory responses [23]. Moreover, similar effects have been stated in studies involving ischemic stroke, where MLT not only reduced pro-inflammatory markers but also enhanced anti-inflammatory markers like IL-10, suggesting a dual action of MLT in this context [24]. The action of MLT is also closely linked to its signaling pathways. For instance, the use of luzindole, an antagonist of MLT receptors, revealed that MLT's anti-inflammatory effects depend on its interaction with the MT2 receptor [25]. This receptor engagement initiates downstream signaling that can influence various cellular responses, illustrating MLT's function as a significant anti-inflammatory agent (Fig. 4).

Additionally, MLT's interplay with cellular pathways involved in autophagy further complicates its therapeutic potential. Jin et al. provide insights into how MLT's influence on the AMPK/mTOR signaling pathway promotes autophagy and cell survival, particularly within endothelial progenitor cells subjected to stress [11]. This indicates that low-dose MLT may foster recovery and enhance healing processes, while higher doses may overwhelm the cellular machinery, triggering death pathways. In essence, the varied mechanisms of MLT action appear intricately tied to dosage, with low doses primarily supporting cell survival and functioning through receptor modulation, while higher doses can induce oxidative stress and apoptosis via ROS-mediated mechanisms. Although such prooxidant effects may be advantageous in targeting malignant cells, they could be detrimental in non-cancerous tissues, thereby highlighting the importance of dose optimization. Indeed, our data suggest that at 100 mg/kg, MLT may lose some of its antioxidant capacity with respect to protein oxidation and lipid peroxidation markers, reinforcing the concept that “more” is not necessarily “better” when it comes to MLT therapy.

### 4.4 Antioxidant safety and prooxidant potential of melatonin

Today, antioxidant safety is one of the most important medical issues in the field of oxidant-antioxidant balance and oxidative stress. It is still unclear which exogenous antioxidants, and their doses should be used for an effective defence and what the safe limit for use is. Disruption of the balance between ROS generation and antioxidants can damage cell components including proteins, lipids and DNA, and oxidative modification of any of these biomolecules can lead to various functional changes and theoretically contribute to disease development [26]. However, several experimental studies suggest that melatonin may exert prooxidant effects under certain conditions [27]. In this respect, it was reported that melatonin significantly increased intracellular ROS and Ca2 + formation, stimulated mitochondrial membrane depolarisation, cytochrome c release, caspase activation, protein phosphorylation and phosphatidylserine externalisation in human platelets [21]. In general, the prooxidant effects of melatonin have been reported only in in vitro cell culture systems, especially in cancer cells. The findings suggest that melatonin at high dose like 100 mg/kg tends to lose its antioxidant properties in terms of protein oxidation and lipid peroxidation markers.

### 4.5 Lipid peroxidation and membrane protection

Another important damage mechanism of ROS is lipid peroxidation. It has been reported in the literature that the highest melatonin concentration is in the cell membrane [12]. In our study, we investigated the protective effect of exogenous melatonin against radiotherapy-induced lipid peroxidation by examining plasma lipid hydroperoxide and malondialdehyde levels. Both lipid peroxidation biomarker levels were significantly higher in the RT group (group 2) compared to the control group. Similar to our study, Kaya et al. reported that plasma malondialdehyde levels decreased in rats treated with 100 mg/kg melatonin [28].

### 4.6 Effects on advanced glycation end products (AGEs)

Melatonin also has the effect of reducing oxidative damage by suppressing the production of advanced glycation end products (AGEs). In a study examining cell recovery when melatonin was given to mouse endothelial progenitor cells, it was reported that apoptosis and cellular dysfunction associated with advanced glycation products decreased[11]. In our study, AGEa were significantly higher in the RT group (group 2) compared to the control group. AGE levels were significantly lower in the low-dose and medium-dose melatonin group compared to the RT group, whereas no significant difference was found at the high dose. Like the literature, melatonin administration was shown to decrease serum AGE levels.

### 4.7 Biochemical findings on antioxidant enzymes

According to the biochemical results of our study, Cu-Zn superoxide dismutase activity, ferric reducing antioxidant levels and total thiol group levels decreased in all groups treated with radiotherapy compared to the control group, while the levels of these parameters increased significantly due to melatonin administration. Low dose melatonin caused an increase in antioxidant parameters, medium dose melatonin and high dose melatonin administration caused an increase in superoxide dismutase activity. Cu, Zn superoxide dismutase activity of the control group was higher compared to all three doses and ferric reducing antioxidant levels were also higher than the highest dose. Due to exposure of rat heart to high dose irradiation, oxidative stress caused significant decreases in the activity of these antioxidant defence enzymes. Similarly, another study demonstrated that irradiation-induced oxidative damage decreased the levels of antioxidant markers catalase, glutathione peroxidase and superoxide dismutase, and found no significant difference in the levels of these biochemical parameters between the control and melatonin groups [17]. According to the first biochemical data obtained in our study, melatonin suppressed the effect of radiotherapy-induced free radicals and increased intracellular Cu, Zn superoxide dismutase activity, ferric reducing antioxidant power and total thiol levels. Therefore, melatonin has been shown to be radioprotective against radiotherapy-induced damage.

### 4.8 Future perspectives and research directions

Macroscopic and histopathological examinations are the gold standard for the evaluation of radiation-induced tissue damage. In our study, inflammatory cell infiltration, perivascular oedema, cardiomyocyte damage and fibrosis were evaluated after rat heart radiotherapy. The anti-inflammatory and protective effects of melatonin against radiotherapy-induced oxidative stress were investigated in the control group, RT alone and RT plus melatonin groups. Although these structures were normal in the control and melatonin-treated groups, significant histopathological changes were observed only in the RT group. In the literature, similar to our study, it has been shown that melatonin administered in addition to radiotherapy significantly reduces oxidative stress in the early stage of rat heart damage and has a cardioprotective effect by increasing histopathological healing [29]. According to biochemical and histopathological results, oxidative stress caused by irradiation increased myocardial tissue damage. However, melatonin administration was found to have anti-inflammatory and antioxidant radioprotective effects on rat heart tissue and serum samples by suppressing early oxidative stress induced by ionising radiation.

## 5 Conclusion

This study demonstrates that melatonin particularly at a dose of 5 mg/kg, offers significant cardioprotective benefits by reducing oxidative damage markers and enhancing antioxidant defenses in rats exposed to thoracic radiotherapy. Our findings highlight the potential of low-dose melatonin as an effective intervention to mitigate radiotherapy-induced cardiotoxicity, suggesting a viable pathway for reducing cardiac complications in radiotherapy patients. Future research should explore the clinical relevance of these results to optimize melatonin dosing in human therapeutic contexts.

## CRediT authorship contribution statement

**Öner Dinçbaş Fazilet:** Writing – review & editing, Writing – original draft, Visualization, Software, Project administration, Methodology. **Karaçam Songül:** Project administration, Investigation, Conceptualization. **Ergen Şefika Arzu:** Writing – review & editing, Writing – original draft, Supervision. **Ülker Melike:** Project administration, Methodology, Investigation. **Şentürk Gözde:** Writing – original draft, Validation, Supervision, Methodology, Investigation. **Ozkan Serbay:** Visualization, Supervision, Formal analysis. **Atukeren Pınar:** Supervision, Resources, Project administration, Data curation. **Yanar Karolin:** Writing – review & editing, Visualization, Formal analysis. **Demi̇r Ecem:** Supervision, Project administration, Methodology, Formal analysis.

## Statements and declarations

All figures submitted have been created by the authors who confirm that the images are original with no duplication and have not been previously published in whole or in part. There is no other contributor. There was no financial support.

## Funding

This research did not receive any specific grant from funding agencies in the public, commercial, or not-for-profit sectors.

## Declaration of Competing Interest

The authors declare that they have no known competing financial interests or personal relationships that could have appeared to influence the work reported in this paper.

## Data Availability

Data will be made available on request.

## Glossary

**1. Melatonin**: A naturally occurring hormone with antioxidant and radioprotective properties, used in this study to mitigate cardiotoxic effects of thoracic radiotherapy.
**2. Protein Oxidation**: A process where proteins are damaged due to oxidative stress, measured as a marker of cellular injury.
**3. Lipid Peroxidation**: Oxidative degradation of lipids, an indicator of membrane damage and oxidative stress.
**4. Advanced Glycation End Products (AGEs)**: Harmful compounds formed through glycation, contributing to oxidative stress and tissue damage.
**5. Cu-Zn Superoxide Dismutase (SOD)**: An antioxidant enzyme that protects cells from damage caused by superoxide radicals.
**6. Iron Ion Reducing Antioxidant Power (FRAP)**: A measure of the antioxidant capacity to reduce ferric ions, used as a marker of redox balance.
**7. Total Thiol Groups**: Compounds containing sulfur groups that act as antioxidants, reflecting the redox status of tissues.
**8. Oxidative Stress**: Imbalance between free radicals and antioxidants in the body, leading to cellular damage.
**9. Non-Enzymatic Redox Homeostasis**: The balance of oxidative and reductive processes maintained by non-enzymatic mechanisms in the body.

## Footnotes

Appendix A Supplementary data associated with this article can be found in the online version at doi:10.1016/j.toxrep.2025.102030.
