# Supplementary material for: Cardiotoxicity prevention in thoracic radiotherapy: The effect of different melatonin doses on the level of oxidation markers -in vivo animal study
Source: Toxicol Rep. 2025 Apr 17;14:102030. doi: 10.1016/j.toxrep.2025.102030 (PMC12047488; doi:10.1016/j.toxrep.2025.102030)
Supplement: Supplementary file 1 — Supplementary material [file mmc1.docx]

**Supplementary Material**

**
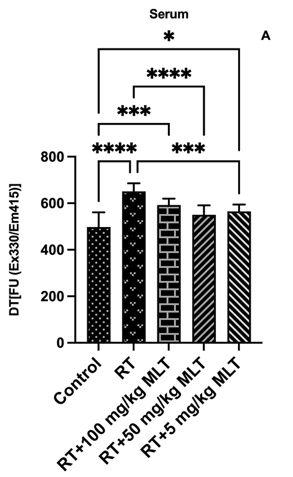

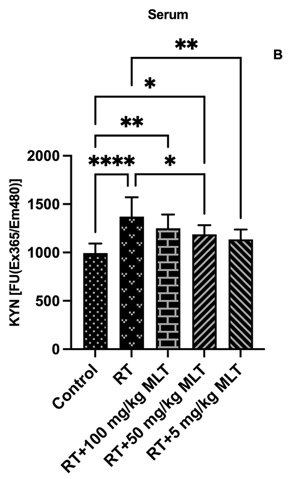

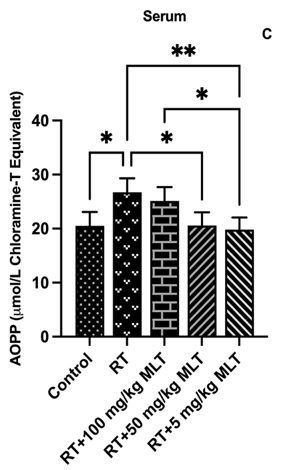

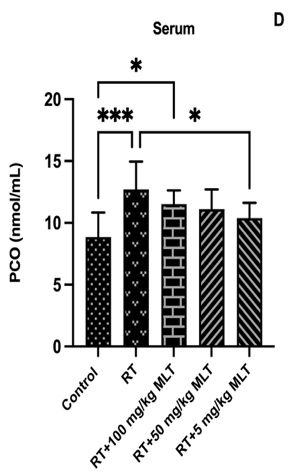
**

**Fig 1.** A-serum Dityrosine (DT), B-Kynurenine (KYN), Advanced Oxidation Protein Products (AOPP) and Protein Carbonyl (PCO) values among groups treated with melatonin (MLT) or saline solution

**(*** p <0,05; ** p<0,01; *** p<0.001; **** p<0.0001)

**
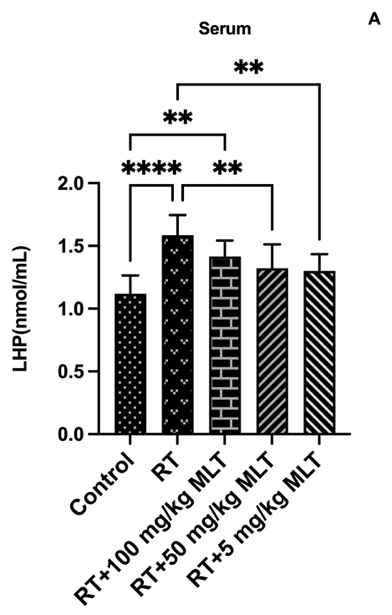

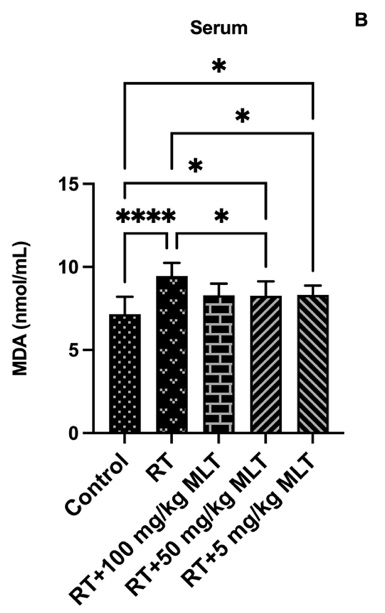
**

**Fig. 2** Plasma Lipid hydroperoxide (LHP), Malondialdehyde (MDH) values of the groups treated with melatonin (MLT) or saline solution

**(*** p <0,05; ** p<0,01; *** p<0.001; **** p<0.0001)

**
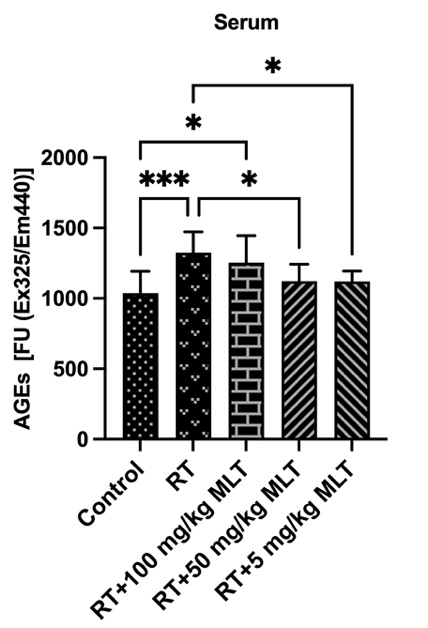
**

**Fig. 3** Serum Advanced Glycation Product (AGE) values of the groups treated with melatonin (MLT) or saline solution

**(*** p <0,05; ** p<0,01; *** p<0.001; **** p<0.0001)

**
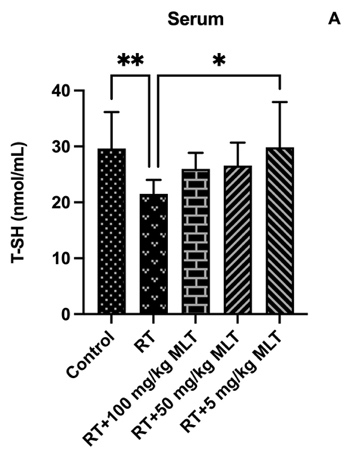

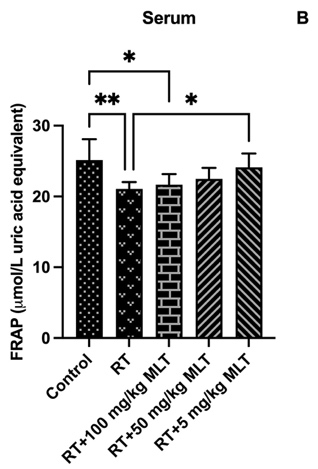

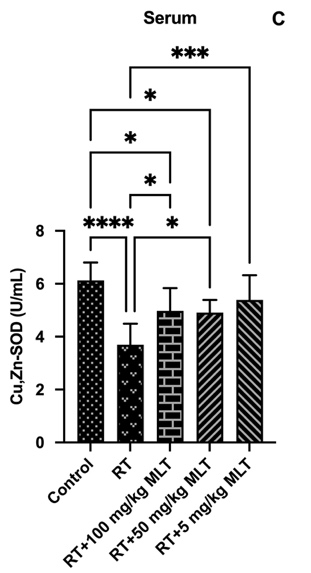
**

**Fig. 4** Serum Total Thiol Groups (T-TG), Ferrous Ion Reducing Antioxidant Power (FRAP) and Cu, Zn-Superoxide Dismutase (Cu, Zn-SOD) values of the groups treated with melatonin (MLT) or saline solution

**(*** p <0,05; ** p<0,01; *** p<0.001; **** p<0.0001)
